# Control of Mosses on Water Flux in an Alpine Shrub Site on the Qilian Mountains, Northwest China

**DOI:** 10.3390/plants11223111

**Published:** 2022-11-15

**Authors:** Zhangwen Liu, Rensheng Chen, Jinxian Qi, Zhiying Dang, Chuntan Han, Yong Yang

**Affiliations:** 1 Qilian Alpine Ecology and Hydrology Research Station, Northwest Institute of Eco-Environment and Resources, Chinese Academy of Sciences, Lanzhou 730000, China; 2 College of Urban and Environmental Sciences, Northwestern University, Xi’an 710127, China; 3 Qilian Forestry and Grassland Administration/Qilian Management Branch of Qilian Mountain National Park (Qinghai), Qilian County 810400, China

**Keywords:** alpine shrub, mosses, Qilian Mountains, water fluxes, seasonal frozen soil, evaporation

## Abstract

Mosses are an important component of the alpine shrub, but little is known about their contribution to ecosystem water and energy exchange, especially potential opportunities for alpine shrub expansion under a warming climate. We studied the role of mosses in alpine shrub evapotranspiration by conducting herb and moss removal experiments with different *Potentilla fruticosa* L. shrub coverage in the Qilian Mountains, Northwest China. The understory evapotranspiration was measured using lysimeters in different shrub coverage (dense shrub cover, medium shrub cover, and thin shrub cover) during the growing season of 2012. The understory evapotranspiration is about 1.61 mm per day in the control treatment (intact moss and herbs) during the growing season, and the evapotranspiration rates differed significantly between canopy covers. We found a 22% increase in evapotranspiration losses after removing the moss layer compared to the control treatment lysimeter with an intact moss layer in the shrub site. This suggests that most of the understory evaporation originated from the organic layer underlying the moss layer. Given this study’s large moss evaporation rates, understory contributions cannot be ignored when interpreting eddy covariance data for the whole alpine ecosystem. Our results show that mosses may exert strong controls on understory water fluxes in alpine shrub meadow ecosystems and suggest that changes in moss cover may have significant consequences for season frozen soil thaw.

## 1. Introduction

Vegetation composition shifts, particularly shrub expansion across the Arctic tundra, are some of the most important and widely observed responses of high-latitude ecosystems to rapid climate warming [1,2]. The increase in relative abundance and cover of deciduous shrub species—a process termed *shrubification* or *shrub encroachment*—during the past decades can be attributed to climate warming with high confidence [3,4]. Although the optimal climate for shrub recruitment has already been passed in some cold regions, accelerated warming and greening trends continue in high latitudes and altitudes [5,6]. In hosting the world’s coldest distribution limits of shrubs, the Arctic and the Tibetan Plateau share some similar ecosystems [7,8]. Shrub expansion remains a common phenomenon in the ecosystem of the Tibetan Plateau [9]. The observed patterns of shrub encroachment suggested that the alpine zones of the southern Tibetan Plateau are shifting from a herbaceous to a shrub-dominated ecosystem [10]. The increase in the cover (+6.8%) and average patch size (+49%) of shrubs has led to 8% of grassland being converted to shrubland in the Qilian Mountains of the northeast Tibetan Plateau [11]. The widespread expansion of woody shrubs into historically herbaceous alpine plant zones constitutes an alteration to vegetation community composition density, distribution, and phenology [12]. After shrub expansion, the growth of herb, moss, and lichen vegetation under the shrub canopy may be affected by changes in the density or extent of shrub cover [13,14]. Consequently, there is a growing need to better understand the effects of alpine shrub expansion and population dynamics on understory vegetation in cold regions.

Mosses are an important component of ecosystems in cold environments, where they often dominate the ground-layer vegetation in forests and shrublands across the alpine biome [15]. Generally, moss biomass on the forest floor tends to effectively buffer soils from variations in atmospheric climate, due to its low thermal conductivity, high porosity, and high water-holding capacity [16]. The moisture exchange at the moss–atmosphere interface is controlled by evaporation losses and precipitation inputs. Because of the lack of stomatal control and distribution as a cushion, mosses fundamentally differ from vascular plants in water exchange with the atmosphere [17]. Mosses can intercept and store large amounts of water from rainfall [18,19], so higher water evaporation rates may be due to the high water-holding capacity of moss or due to their large surface area [20]. This makes mosses an important contributor to water loss in many cold ecosystems [21,22]. Mosses have received attention for their importance in regulating soil moisture in cold forest ecosystems [21]. Many studies have found that moss can evaporate water at higher rates than adjacent bare soil surfaces [23,24]. Among plant types, evapotranspiration (ET) from moss-covered and inundated areas were more than twice that from other plant types at a field site near Barrow, Alaska, USA [25]. Moss evaporation rates depended strongly on the openness of the forest and, to a lesser degree, on the plant canopy density in a black spruce forest in Fairbanks, Alaska [21]. Bridgham et al. (1999) observed larger evapotranspiration in moss-dominated bog plots than in sedge-dominated fen plots [25]. However, Rocha and Shaver (2011) [26] found that reduced moss cover caused by fire increased soil evaporation. In the Siberian tundra, understory evapotranspiration under shrubs increased with the removal of the green moss layer, which suggested that most of the understory evapotranspiration originated from the organic soil layer underlying the green moss layer [13]. At the Canadian bog site, soil moisture beneath Sphagnum communities’ cushions was 5–14% higher than adjacent bare cutover peat [27]. Moss can also achieve limited homeothermy through soil water evaporation, similar to the vascular plant through transpiration, and the temperature threshold was around 30 °C in South China [22]. In theory, moss is unable to control the evaporation process, and there are no stomata that regulate water transpiration [28]. In addition, the internal water transport system of moss is poorly developed [29]. Experimental observations confirm that moss cover does affect evapotranspiration in cold region ecosystems [13,21]. So far, the mechanisms by which mosses control evapotranspiration are not well understood [30].

Evaporation of the moss surface results in an energy loss from the soil surface because of the latent heat flux involved with vaporization in the permafrost region. Mosses may also cool the soil by water evaporation from their surface [13]. Shrub cover influences water and heat flux exchange of the moss layer [29]. On the one hand, moss growth may be reduced directly by higher air temperature because of the relatively low temperature optima of mosses for photosynthesis, and reduced indirectly by increased shading by shrub canopy and associated leaf litter [13]. On the other hand, except for the negative effect, shading of the shrubs has also favored moss growth by alleviating the prate inhibition of photosynthesis [14,31]. Therefore, increasing vascular plant cover density may also decrease the evaporation of the moss layer [13,21,32]. Evaporative water loss and drying of the moss layer tend to progress from the surface downwards, with a more stable microclimate deeper within the moss layer [33]. Higher moss biomass has been linked to greater moisture retention by mosses, so the deeper moss layer in late successional stands may have more potent effects on the ET [34]. There is no significant effect of shrub canopy density on understory evapotranspiration, but denser shrub canopy could reduce the ground heat flux in Northern Siberia [13]. An increased understanding of the moisture dynamics and water exchanges between moss cushions and expansive shrubs is required. Despite these potentially strong effects of mosses on water flux, very little research has been conducted on them in seasonal frozen soil regions [20]. They are poorly represented in models and experiments used to predict the effects of moss on water flux [21,30]. Thus, it would be worth conducting more experiments to understand how moss controls soil evaporation in seasonal frozen soil regions.

Given these uncertainties, one meaningful way forward is to combine field observations with experimental manipulations to examine the hydrological effects of moss in the changing environment. Our study aimed to understand the role of mosses in alpine shrub evaporation. The present study of the influence of mosses on evapotranspiration in an alpine shrub site in the Qilian Mountains of China was designed to respond to the following: (1) What is the contribution of mosses to shrub evapotranspiration? (2) What is the effect of shrub canopy cover on moss controls on evapotranspiration?

## 2. Results

### 2.1. Evapotranspiration on Different Shrub Cover

Plant cover differed distinctly between the DSC, MSC, and TSC plots, especially shrub cover (Table 1). Although shrub cover was highly variable, there was minimal variation in moss cover within the plots. Significant differences (*p* < 0.05) were found between shrub plots with different canopy covers (Table 1). There were also more tall shrubs in the dense shrub than in the open shrub plot, which was included in the aboveground biomass, basal stem diameter, and leaf area index. Fewer understory herbs were covered in the dense shrub than in the open alpine shrub meadow (Table 1).

**Figure 1:**
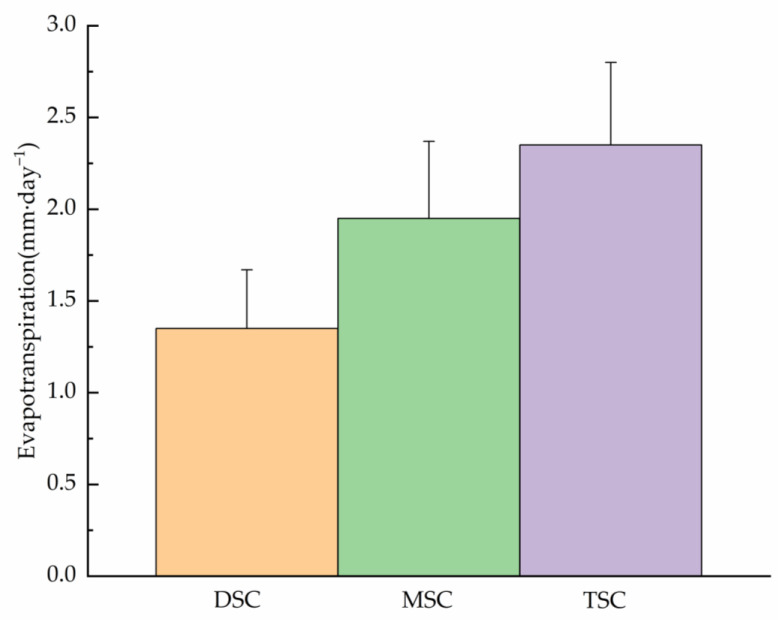
Daily average evapotranspiration rates between lysimeter (intact herbs and moss) under different shrub canopy coverage during the entire period of measurement (102 days) of 2012.

The shrub cover had a powerful significant effect on evapotranspiration under the shrub canopy (Figure 1, Table 2). Rates of evapotranspiration differed significantly between canopy covers (Table 2). Evaporation rate in the TSC, MSC, and DSC plot were 2.35, 1.95, and 1.35 mm day^−1^, respectively. The daily evapotranspiration rate in the DSC plot was only 70% of that in the MSC plot. The mosses evaporated in the TSC plot on average 1.74 times more than in the DSC Plots. The surrounding vegetation cover and moss treatments also significantly affected moss evaporation (Table 2). Moss evaporation was larger when the lysimeters were surrounded by relatively open herb vegetation than by denser and taller vascular plant vegetation (Figure 1).

**Figure 2:**
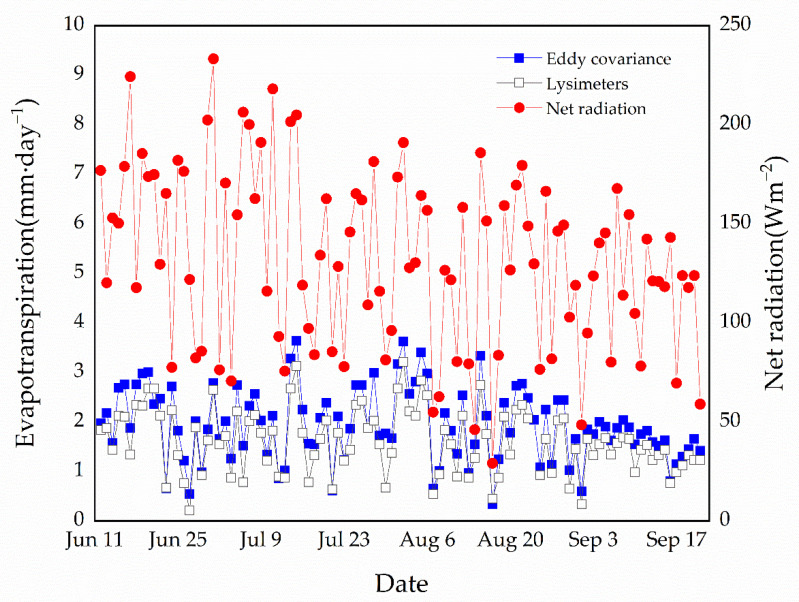
Evapotranspiration rates between eddy covariance technique for alpine meadow and lysimeter (intact herbs and moss) during the growing season in 2012. The blue line and solid squares represent daily average tundra evapotranspiration measured by the eddy covariance technique. The black line and hollow squares represent daily average evapotranspiration rates measured by lysimeters with intact graminoid and moss vegetation (*n* = 9 lysimeters). The red line and solid circles represent the daily average alpine meadow net radiation, as measured by a radiometer on the eddy covariance tower.

We compared daily alpine meadow evapotranspiration, as measured by the eddy covariance system, with understory evapotranspiration rates measured by the lysimeters (intact herbs and moss) (Figure 2). Lysimeter evapotranspiration refers to the completion of intact herbs and moss. There is a strong correlation between daily average understory lysimeter evapotranspiration and alpine meadow evapotranspiration measured by eddy covariance (r = 0.95, *p* < 0.001, *n* = 102 days). The alpine meadow rates range between 0.4 and 3.6 mm day^−1^, and the average evapotranspiration rate is 1.94 mm day^−1^. The understory evapotranspiration rates range between 0.2 and 3.2 mm day^−1^ and the average evapotranspiration rate is 1.61 mm day^−1^. We measured a relatively large variability in daily average understory evapotranspiration (intact graminoid and moss treatment), which was closely linked to daily variations in net radiation (r = 0.65, *p* < 0.05, *n* = 102 days).

### 2.2. Evapotranspiration on Different Treatments

**Figure 3:**
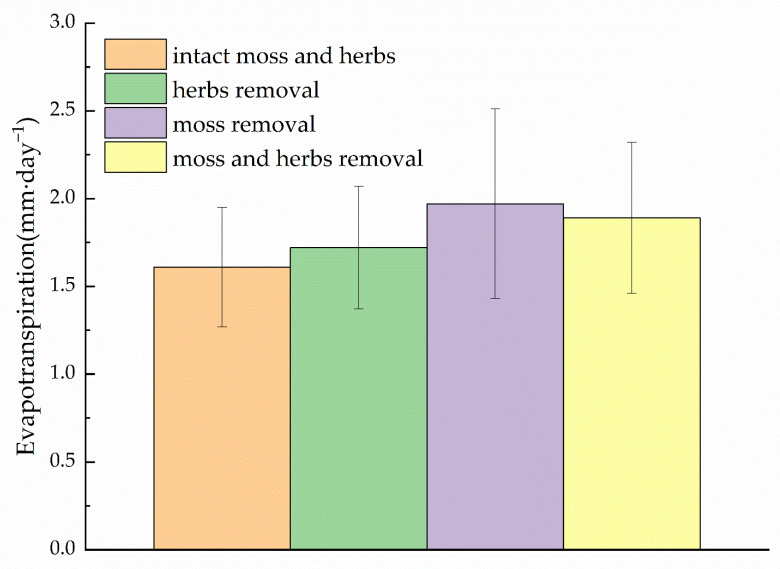
Comparison of daily average evapotranspiration rates between lysimeter treatments between 12 June and 21 September of 2012. Lysimeter treatments consisted of four groups: orange bar, with intact herbs and moss vegetation; green bar, herbs with moss removed; purple bar, moss with graminoid removed; and yellow bar, with moss and graminoid removed. Data are mean values ± SE (*n* = 9 lysimeters). Significances of treatment effects of moss removal and graminoid removal on evapotranspiration are provided in Table 2.

The mixed-model analysis showed that evapotranspiration rates differ significantly between the different lysimeter treatments (Table 2). The highest evapotranspiration rate was found in the moss layer removal treatment, with a daily evapotranspiration rate of 1.97 mm per day, and the lowest in the control treatment (intact moss and herbs), with 1.61 mm per day. Thus, two lysimeter treatment groups could be distinguished based on their evapotranspiration rates: lysimeters with and without moss layer (*p* < 0.001, *n* = 18). We found a 22% increase in evapotranspiration losses after removing the moss layer compared to the control treatment lysimeter with an intact moss layer in the shrub site in the Qilian Mountains (Figure 3). This indicates that the living moss layer is a barrier to water exchange between the underlying brown organic layer and the atmosphere. This suggests that mosses may also inhibit evapotranspiration in the understory despite their lack of stomatal control, which is considered free evaporation. The shading of the organic layer by the intact green moss layer may have limited the total evapotranspiration (moss and organic soil evaporation) from the understory. This is mainly because the vapor pressure gradient between the lower moss layer and the air is reduced, thus reducing evapotranspiration mainly from the lower organic soil layer. In addition, evaporation from green moss layers may be limited by soil water availability from organic soils.

## 3. Discussion

### 3.1. Control of Moss on Evapotranspiration in Shrub Site

**Figure 4:**
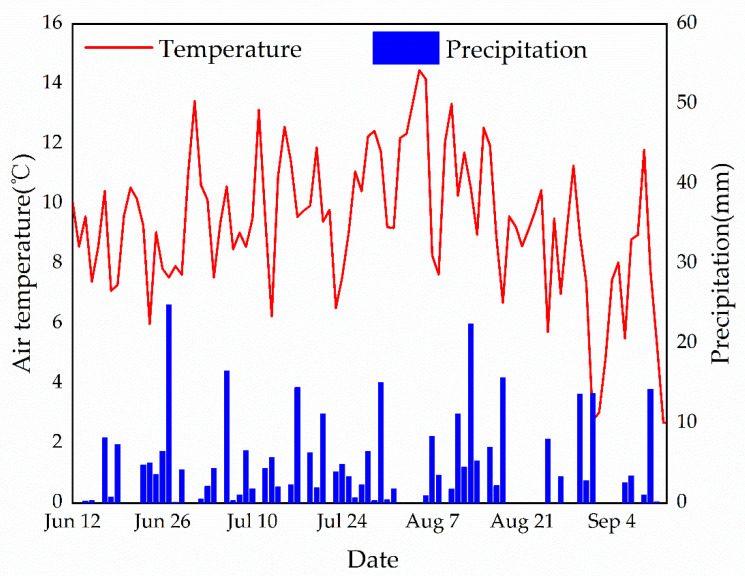
The air temperature and precipitation in the shrub site during the experiments’ period in 2012.

Although mosses lack stomatal control and their surfaces are thought to evaporate freely, mosses may also inhibit evapotranspiration in the understory of alpine shrubs in the Qilian Mountains. This suggested that the living moss layer serves as a barrier to water exchange between the underlying brown organic layer and the atmosphere (Figure 3). From the physiological-ecological point of view, mosses belong to variable water plants [16]. Although mosses are small, they often form large clumps or mat-like communities with interlocking branches and leaves forming a large number of capillary pores. They possess the characteristics of fast water absorption and large water storage [20]. Because the moss vegetation has no stomata, when the moss water content is too low, the moss insulation increases due to the increase in air composition, which reduces the moss absorption of water from the soil [33]. When there is sufficient water input, the moss will quickly absorb a large amount of water again, and the water-holding rate will reach the maximum [20]. The mosses could absorb water that exceeds its weight from 386.94% to 782.51% in the laboratory. The maximum water-holding rate belongs to the *P. fruticosa* shrub in the Hulu catchment [20]. The weather conditions during our experiments were relatively wet. The frequent precipitation events ensured that the moss layer remained moist, so understory evapotranspiration was probably not limited by insufficient moisture supply in the study site (Figure 4) [35]. At the same time, the temperature was relatively high throughout the growing season of 2012. Therefore, in a water deficit, alpine shrub mosses can absorb a large amount of water, but when water is sufficient, the moss can be saturated with water quickly. Evaporation reacted strongly to experimental water additions, indicating that precipitation frequency is an important factor, in addition to microclimate, for moss species [21]. Our experimental results also support this argument, where the frequency and amount of precipitation are high, and understory evapotranspiration is reduced due to low net radiation.

Soil properties also had an important effect on evapotranspiration. There was no difference in soil bulk density between the three sample sites before and after the experiment (Table 3). Although soil moisture content did not differ at the beginning of the experiment, soil moisture increased significantly in the DSC and MSC plots after the experiment. The volumetric water content of green moss in the lysimeters started at an average of 54% and was 76% at the end of the experiment. Especially in the DSC plots, the moss water content increased significantly when evaporation was not limited by water, similar to Blok et al. Due to the canopy cover, the moss moisture content of DSC was slightly higher than other plots at the beginning of the experiment. At the end of the experiment, the forest understory received less net radiation due to the high canopy cover in the DSC plots [36]. Therefore, the moss surface evaporation was slight, and the water content was relatively high. The water flux mainly evaporated from the moss surface at this time, while the soil layer evaporated less, which is similar to the results of Blok et al.’s experiments in Northern Siberian [13].

The understory evapotranspiration is about 1.61 mm per day in the control treatment lysimeters (intact moss and herbs). This rate is lower than the evapotranspiration rate measured with lysimeters by Yang et al. (2017) in an alpine meadow at 3000 m elevation in the same catchment [37]. They observed the mean daily ET of 2.0 mm per day in the unfrozen period. The low ET in our study site may be due to two reasons. On the one hand, our study site is at a higher altitude than their study site (3232 m vs. 3000 m a.s.l.), and the air temperature was cooler at the shrub meadow site. On the other hand, the alpine meadow they measured was not covered by a shrub canopy, and the meadow received higher solar radiation than the meadow under the shrub. The dominant factor affecting ET in the unfrozen period was net radiation, which is consistent with this study’s findings. Our values are comparable with moss understory evapotranspiration rates measured in cold environments. Our result is consistent with Heijmans et al. [21] in a boreal black spruce forest in Alaska, where vegetation structure influenced understory evapotranspiration. There were differences in understory evapotranspiration in the Siberian tundra at different shrub densities, but the differences were not significant [13]. Several studies on the sub-alpine Qinghai spruce forests in the northeastern Tibetan Plateau revealed that rainfall significantly affected the energy flux distribution and evapotranspiration. During the growing season, the soil water content was low but not easily lost [38]. A long-term growing season flux observation of humid alpine shrubs in the northeastern Tibetan Plateau revealed that alpine shrubs served as a water source during the growth period, and seasonal changes in actual evapotranspiration were determined by net radiation and water vapor pressure loss. At different growth stages, there was a significant difference in response to water vapor exchange [39].

Our experiments focused on the role of mosses in the evapotranspiration in an alpine shrub site. There is an increase in evapotranspiration losses after removing the moss layer in the shrub site. This suggests that moss acts as a physical barrier to soil moisture, and most of the understory evaporation originated from the organic layer underlying the moss layer. However, plots, in terms of their ecophysiological data on mosses, are lacking in this experiment. Therefore, this experiment does not answer whether mosses are active or passive to transpiration. Follow-up studies should incorporate ecophysiological properties of mosses, such as water potential, moss species, ecological and anatomical features, etc. [17]. This will allow us to better understand the water transport processes within mosses and distinguish how mosses affect evapotranspiration (active or passive). In addition, evapotranspiration experiments should add measurements of soil temperature and heat flux [13,31]. These complementary experiments can provide a greater understanding of how mosses control water and heat fluxes in alpine ecosystems.

### 3.2. The Cooling Capacity of Moss Evapotranspiration

Because of their low thermal conductivity, high porosity, and high water-holding capacity, mosses can reduce soil temperature by restricting energy transfer into the soil from variations in atmospheric climate [16]. The cooling effect of soil temperature by moss may be due to its ability to accelerate soil water evaporation. Most vascular plants achieve limited homeothermy by transpiration, but moss has no organs which can regulate transpiration [22]. Moss can also achieve limited homeothermy through soil water evaporation, similar to the vascular plant through transpiration. This homeothermy led to the decoupling of soil temperature and near-surface air temperature [22]. Mosses play an important role in permafrost stability by buffering surface soils and permafrost from fluctuating air temperatures. In the growing season, surface soil temperatures are negatively related to the thickness of organic soil layers [40]. Moisture conditions of the moss tissue thus likely determine whether mosses may achieve soil cooling during summer primarily by thermal insulation or evaporation [13]. Mosses exert strong control over tundra soil microclimate, mediating the exchange of water and energy between soil and atmosphere in permafrost. In contrast, it has been suggested that shading by shrub canopies may decrease summer soil temperatures in permafrost [41]. Both observational and manipulative studies have quantified the importance of mosses to ground heat flux [13].

The large moss evaporation rates suggest a potential cooling effect of mosses in the cold ecosystem. The changes in soil moisture regimes will affect decomposition with predicted increased evapotranspiration rates under shrubs, drying soils and potentially leading to microbial moisture limitations [40]. Moreover, modeling studies incorporating thick organic soils have shown their importance in permafrost dynamics [42]. While some moss species possess high moisture retention, in general, the porosities of organic soil layers are higher than mineral soils [43]. The sensitivity experiments confirmed that a moss layer on the ground surface resulted in a warmer winter soil temperature and cooler summer soil temperature compared with a moss-free case [40]. The increase in the moss thickness enhanced the insulation effect of moss on the soil. The role of moss cover on soil temperature was substantially stronger in summer than in winter.

### 3.3. Shrub Expansion and Moss Evapotranspiration

Increases in shrub biomass, cover, and abundance (*shrubification*) have been observed in many Arctic, high-latitude, and alpine tundra ecosystems using repeat photography, long-term ecological monitoring, and dendrochronology, over the past century [1]. Shrub encroachment occurs in arid grasslands worldwide, and the interactions and feedbacks between soil moisture/temperature and woody plants remain poorly investigated in arid and semi-arid regions [41]. Climate warming will result in alpine shrub expansion towards the interior of the Tibetan Plateau, with an average center shift of 309 km under the RCP4.5 scenario with a warming of 1.5 °C. Alpine shrubs will replace a proportion of alpine meadow, causing it to shrink by ~6.8% under RCP4.5 with a warming of 1.5 °C and by ~15.5% under RCP8.5 with a warming of 2 °C [44]. Despite these potential opportunities for alpine shrub growth under a warming climate, several abiotic and biotic factors may constrain shrub encroachment [45]. Increasing temperatures will move the vegetation boundaries northwestward, and increases in precipitation will enhance this trend. Turetsky et al. [40] 2012 reported that moss benefits more from colder temperatures than vascular plants. Increased shrub cover could reduce moss biomass, a vital soil insulator. Thus, the loss of the moss layer may alter soil temperatures, active layer depths, and soil evaporation rates [13]. An increasing tree density induces a range of additional feedback mechanisms that regulate evaporative losses under a changing canopy density [46].

On average, alpine meadow evapotranspiration exceeded understory evapotranspiration, as measured by lysimeters with intact graminoids and mosses, by 21%, especially during sunny days with relatively high net radiation (Figure 2). Our findings are contrary to the mosses of Blok et al. in the Siberian tundra shrub site, where understory evapotranspiration is higher than tundra evapotranspiration. At their site, the *Betula nana* shrub canopy is only 10–20 cm tall, which makes it likely that the understory contributes greatly to whole-ecosystem evapotranspiration. However, at our study site, the *P. fruticosa* shrub canopy is 40–80 cm tall, and the canopy reduces the rainfall and incoming radiation. The canopy structure of alpine shrubs is the most important factor influencing the water-holding capacity of mosses under shrubs [20]. Canopy is also an important factor influencing understory water flux loss. Therefore, shrub canopy cover had a significant effect on evapotranspiration. Rates of evapotranspiration differed significantly between canopy covers (Table 2). If the open canopy architecture and relatively high moss coverage were substantially reduced in the colonizing shrub at lower elevations, then the expansion of alpine shrubs into meadow areas would exert little influence on regional energy exchange and water supply. In summary, shrub expansion into areas of meadows could improve the magnitude and stability of the regional water supply by reducing ET loss and enhancing the soil water-holding capacity [47].

## 4. Materials and Methods

### 4.1. Study Site Description

The field experiments were conducted in an alpine shrub site in Hulu catchment with an area of ~23.1 km^2^ (38°12′–38°17′ N, 99°49′–99°54′ E, 2960–4800 m elevation) in the Qilian Mountains of China [48,49]. The geology of Hulu is dominated by bedrock in the high mountains, including Lower Ordovician metamorphic and volcanic rocks, interbedded metamorphic rocks, and slates [50]. The Hulu catchment’s landscapes vary and exhibit a gradient distribution with increasing elevation, including grassland, meadow, shrub, forest, marshy meadow, cushion plant, etc. *Picea crassifolia* Kom. and *Sabina przewalskii* Kom are the main tree species in the catchment. Shrubs are mainly distributed on the region’s shady, semi-shady, and semi-sunny slopes. The main shrub species are *Potentilla fruticosa* L., *Caragana jubata* Poir., *Salix cupularis* Rehd., and *Salix oritrepha* Schneid. Among them, the *P. fruticosa* shrub is not only the dominant species in this catchment but also the dominant shrub species in the Qilian Mountains. Herbs and mosses dominate the understory of shrubs. Herbs include *Ploygonum sphaerocephalum*, *Kobresia humilis* (Villars) Foiri., *Stellera chamaejasm* Linn., *Potentilla polyphylla* Wall. ex Lehm., *Polygonum viviparum* L., etc. A Geophytia moss layer almost completely covers the ground with *Distichium brevisetum* C. Gao, *Distichium bryoxiphioidium* C. Gao, and *Ditrichum flexicaule* (Schwaegr.) Hamp. Due to the high altitude and low temperatures, this region’s mosses are mostly cold-tolerant species. The site is covered by a wide variety of habitat types, with severe cold and precipitation throughout the year, forming a relatively humid environment suitable for the growth of moss vegetation. Soils are generally classified as Gelisols, Entisols, Inceptisols, Mollisol, Histosols, etc. Soils are characterized by an organic-rich surface layer underlain by a gravel layer.

A cryospheric hydrometeorology observation system (CHOICE) was established in 2008 in the Hulu catchment [48,49]. The regional climate is strongly continental, with a mean January temperature of −18.4 °C and a mean July temperature of 19.0 °C from 2008 to 2021. The mean annual air temperature is −0.3 °C, and the average annual precipitation is 599.8 mm, with approximately 82% of the precipitation falling as rain from June to October. The mean annual relative humidity is 54.2%, wetter during July and August than in other months. The growing season for vascular plants lasts from late May to late September [51].

### 4.2. Experiment Design

#### 4.2.1. Filed Lysimeter Evapotranspiration Experiments

As the dominant species in the Qilian Mountains, the *P. fruticosa* shrub was selected for conducting field lysimeter evapotranspiration experiments. The experiment plots used in this study were located within these *P. fruticosa*-dominated patches at 3232 m above sea level (a.s.l.). We selected *P. fruticose* shrub experiment plots (5 m × 5 m) with three types of coverage: dense shrub cover (DSC, 75% canopy coverage), medium shrub cover (MSC, 40% canopy coverage), and thin shrub cover (TSC, 25% canopy coverage). Plant species cover in all plots was measured during the summer of 2012 using the quadrat sampling method (Table 1) [52]. Leaf area index (LAI) was determined using a CI-110 canopy analysis system (CID, USA). The average height and basal stem diameter of 20 shrubs were measured by straightedge and vernier caliper (Table 1).

**Figure 5:**
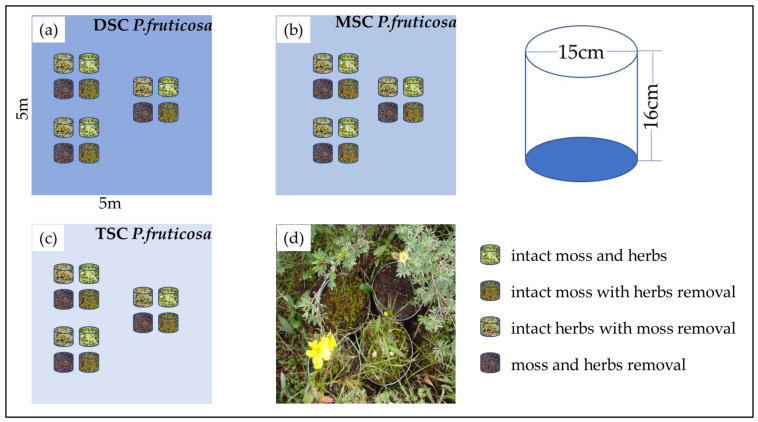
The design graph of the lysimeter evapotranspiration experiment. Three repeated treatment groups were set in each plot: dense shrub cover (DSC) (**a**), medium shrub cover (MSC) (**b**), thin shrub cover (TSC) (**c**), and a photograph of lysimeters under a *P. fruticosa* canopy (**d**). Four lysimeters were used with the following treatments: intact moss and herbs, intact moss with herb removal, intact herbs with moss removal, and moss and herb removal.

The understory evapotranspiration was measured using lysimeters with a height of 16 cm and a diameter of 15 cm. The lysimeters were created by placing vegetation columns with moss and herbs vegetation inside iron containers with closed bottoms. Vegetation and attached soil columns were cut from shrub meadow patches between the plots to exclude the possibility of differences in moss conditions before the start of the experiment [13]. The four lysimeters were set up as a treatment group per plot, including intact herbs and moss, moss removal and leaving herbs intact, herb removal and leaving moss intact, and herb and moss removal. The experimental design was thus four lysimeters as a group in each shrub canopy coverage habitat (Figure 5). Three repeated treatment groups were set in each plot, resulting in 36 lysimeters in this experiment. The top of the green moss and herbs was removed by hand plucking until the organic layer underneath became visible (Figure 5). Lysimeters were weighed by an electronic weighing scale (0.1 g precision) every evening between 12 June and 21 September 2012, and then evapotranspiration was calculated as mm water per day. Daily evapotranspiration rates were corrected for water inputs by precipitation, recorded by an electronic rain gauge at the same site.

At the beginning and end of the experiment, the 100 cm^3^ cutting ring was used to collect soil samples from the 0–15 cm soil layer at three plots, respectively. The drying method determined soil physical properties such as water content and bulk density in the laboratory. In each plot, three samples of 1 m^2^ around each lysimeter group were chosen to harvest and collect the shrub’s aboveground biomass. The fresh weights of stems and leaves of the shrubs were clipped and weighed. In addition, four 1 m by 1 m subsamples were selected at each shrub plot’s corners. The species, number, average height, and cover of the mosses and herbs in the subsamples were all carefully investigated. A 1 m by 1 m herb sample was also set up, and the fresh weight of the aboveground parts of herbs and mosses by species was harvested using the same procedure as the shrub samples. The plant samples were dried, using a drying method, to a consistent weight, at 70 °C for four days before weighing for dry weight in the laboratory. The fresh weight ratio to the dry weight of the samples in the harvested samples was used to calculate the aboveground biomass per unit area of the shrubs, herbs, and mosses (Table 1). Volumetric green moss water content was determined from the difference between fresh and dry weight [20]. Moss biomass was not determined per species, but the identities of the dominant moss species in the lysimeters were noted.

#### 4.2.2. Eddy Covariance Evapotranspiration

In addition to daily evaporative losses measured by lysimeters, larger-scale alpine meadow ecosystem evapotranspiration rates (W·m^−2^) were measured by eddy covariance instrumentation. An eddy covariance tower was installed at a distance of 50 m from our experimental sites and at a measurement height of 5.8 m above ground in September 2011 (EC150, Campbell Scientific Inc., Logan, UT, USA) [49]. EC150 is an open-path analyzer specifically designed for measuring eddy covariance flux. Combined with the CSAT3A sonic anemometer, these two components of an open-path eddy-covariance system simultaneously measure carbon dioxide, water vapor, air temperature, barometric pressure, three-dimensional wind speed, and sonic air temperature. The half-hourly latent heat flux values measured by eddy covariance were converted to evapotranspiration rates and then were summed to daily evapotranspiration values. Net radiation was measured by four-component radiation (CNR1, Kipp & Zonen, Delft, The Netherlands). The measurements included short- and long-wave upwelling and downwelling radiation. Both variables are measured at the same height as the eddy covariance instrumentation.

### 4.3. Data Analysis

To determine the effects of shrub cover and removal treatments on evaporation flux, we used variance analysis (ANOVA) to analyze shrub coverage and moss treatment as independent (fixed) factors. The design of the moisture experiment was a fully crossed factorial with three shrub coverage and four removal treatments, with seven replicates of all combinations. Mixed-model analysis was then applied to lysimeter evapotranspiration data with lysimeter treatment (moss and herb removal) and shrub canopy coverage (DSC, MSC, or TSC of *P. fruticosa* canopy) as explanatory variables and plot as a random variable. To void temporal pseudoreplication, measurement time (lysimeters were weighed on 102 dates between 12 June and 21 September 2012) was severed as a repeated variable. Daily lysimeter evapotranspiration rates were compared to daily net radiation, air temperature, and daily meadow evapotranspiration by calculating Pearson’s correlation coefficients (*r*). Analyses of evapotranspiration data were performed using SPSS 22.0 for Windows.

## 5. Conclusions

The understory evapotranspiration is about 1.61 mm per day in the control treatment lysimeters (intact moss and herbs) during the growing season in the *P. fruticosa* shrub site in the Qilian Mountains, Northwest China. We found a 22% increase in evapotranspiration losses after removing the moss layer compared to the control treatment lysimeter with an intact moss layer in the shrub site. Understory evapotranspiration increased with the removal of the moss layer, suggesting that most of the understory evaporation originated from the soil layer underlying the moss layer. The shrub cover had a powerful significant effect on evapotranspiration under the shrub canopy. Rates of evapotranspiration differed significantly between different shrub canopy covers. The surrounding vegetation cover and moss treatments also significantly affected moss evaporation. Moss evaporation was larger when the lysimeters were surrounded by relatively open herb vegetation than by denser and taller shrubs vegetation. As the moss evapotranspiration differed in response to shrub cover conditions, the control of the moss layer on water flux should also be taken into account in calculating the regional water balance. Given this study’s large moss evaporation rates, understory contributions cannot be ignored when interpreting eddy covariance data for the whole shrub meadow ecosystem. Our results show that mosses may exert strong controls on understory water fluxes in alpine shrub meadow ecosystems and suggest that changes in moss cover may have significant consequences for seasonal frozen soil thaw. Further research is required to characterize shrub expansion in the alpine biome and effectively quantify moss’s evaporation regulation.

## Figures and Tables

**Table 1:** Plant community structure and biomass in the DSC, MSC, and TSC *P. fruticosa* shrub plots during the growing season of 2012.

Plot Type	DSC *P. fruticosa*	MSC *P. fruticosa*	TSC *P. fruticosa*
	75% Coverage	50% Coverage	25% Coverage
Plant cover (%)			
*P. fruticosa* shrub	75.1 ± 4.6 a	49.9 ± 5.3 b	25.3 ± 4.2 c
Herbs	18.8 ± 1.7 a	33.4 ± 5.9 b	41.2 ± 5.1 b
Moss	81.4 ± 3.4 a	82.6 ± 4.2 ab	83.3 ± 6.1 b
Leaf area index	2.57 ± 0.37 a	1.40 ± 0.28 a	0.85 ± 0.23 b
Aboveground biomass (g·m^−2^)			
*P. fruticosa* shrub	2254.86 ± 245.23 a	1832.32 ± 143.56 b	1248.34 ± 112.63 c
Herbs	295.32 ± 65.45 a	389.90 ± 55.32 b	405.43 ± 65.22 b
Moss	450.56 ± 43.45 a	434.19 ± 45.78 a	446.76 ± 50.87 a
Shrub height (m)	0.81 ± 0.23 a	0.76 ± 0.21 a	0.54 ± 0.17 b
Shrub basal stem diameter (cm)	0.54 ± 0.11 a	0.43 ± 0.09 ab	0.35 ± 0.07 b

Plant cover data are means ± SE (*n* = 3 plots) per plot type. Shrub height and basal stem diameter data are mean ± SE (*n* = 20 shrubs). Different letters (a, b, and c) indicate significant differences (Tukey test, *p* < 0.05) between the plots.

**Table 2:** Results of mixed-model analysis of variance of moss evapotranspiration by different plots and lysimeter treatments for the entire measurement period.

Explanatory Variables	df	*F*	Sig.
Plot type	2	8.215	0.038
Herb removal	1	3.455	0.238
Moss removal	1	26.38	<0.001
Plot type × herb removal	2	0.966	0.458
Plot type × moss removal	2	0.124	0.654
Herbs removal × moss removal	1	2.341	0.157
Plot type × herb removal × moss removal	2	5.542	0.087

**Table 3:** Soil bulk density, soil moisture content, and moss moisture content in lysimeters at the start and end of experiments on different shrub plots.

Plot Type	DSC *P. fruticosa*	MSC *P. fruticosa*	TSC *P. fruticosa*
Soil bulk density (g/cm^3^)			
Start of experiments	0.56 ± 0.11 a	0.56 ± 0.09 a	0.54 ± 0.08 a
End of experiments	0.55 ± 0.09 a	0.54 ± 0.08 a	0.53 ± 0.08 a
Soil moisture content (%)			
Start of experiments	38 ± 4 a	36 ± 3 a	35 ± 4 a
End of experiments	69 ± 4 a	64 ± 5 a	58 ± 5 b
Moss moisture content (%)			
Start of experiments	53 ± 6 a	52 ± 5 a	49 ± 5 a
End of experiments	82 ± 5 a	76 ± 4 b	72 ± 4 b

Data are means ± SE (*n* = 12). Different letters (a, b) indicate significant differences (Tukey test, *p* < 0.05) between the plots.

## Data Availability

The data for this study are available by contacting Z.W.L. at zwliu@lzb.ac.cn.
